# Neoadjuvant cisplatin and paclitaxel modulate tumor-infiltrating T cells in patients with cervical cancer

**DOI:** 10.1007/s00262-019-02412-x

**Published:** 2019-10-15

**Authors:** A. Marijne Heeren, Iske F. van Luijk, Joost Lakeman, Noëlle Pocorni, Jeroen Kole, Renée X. de Menezes, Gemma G. Kenter, Tjalling Bosse, Cornelis D. de Kroon, Ekaterina S. Jordanova

**Affiliations:** 1 Department of Obstetrics and Gynecology, Center Gynecological Oncology Amsterdam (CGOA), Amsterdam UMC, VU University Medical Center Amsterdam, De Boelelaan 1117, 1081 HV Amsterdam, The Netherlands; 2 Department of Medical Oncology, Cancer Center Amsterdam, Amsterdam UMC, VU University Medical Center Amsterdam, Amsterdam, The Netherlands; 3 Laboratory for Physiology, Institute for Cardiovascular Research, Amsterdam UMC, VU University Medical Center Amsterdam, Amsterdam, The Netherlands; 4 Department of Epidemiology and Biostatistics, Amsterdam UMC, VU University Medical Center Amsterdam, Amsterdam, The Netherlands; 5 Department of Pathology, Leiden University Medical Center, Leiden, The Netherlands; 6 Department of Gynecology, Leiden University Medical Center, Leiden, The Netherlands

**Keywords:** Cervical cancer, Neoadjuvant chemotherapy, Cisplatin, Paclitaxel, Tumor microenvironment, T cells

## Abstract

Resistance to chemotherapy is widely recognized as one of the major factors limiting therapeutic efficacy and influences clinical outcomes in patients with cancer. Many studies on various tumor types have focused on combining standard-of-care chemotherapy with immunotherapy. However, for cervical cancer, the role of neoadjuvant chemotherapy (NACT) on the local immune microenvironment is largely unexplored. We performed a pilot study on 13 primary cervical tumor samples, before and after NACT, to phenotype and enumerate tumor-infiltrating T-cell subpopulations using multiplex immunohistochemistry (CD3, CD8, FoxP3, Ki67, and Tbet) and automated co-expression analysis software. A significant decrease in proliferating (Ki67^+^) CD3^+^CD8^−^ T cells and FoxP3^+^(CD3^+^CD8^−^) regulatory T cells was observed in the tumor stroma after cisplatin and paclitaxel treatment, with increased rates of cytotoxic CD8^+^ T cells, including activated and CD8^+^Tbet^+^ T cells. No effect was observed on the number of tumor-infiltrating T cells in the cervical tumor microenvironment after treatment with cisplatin only. Therefore, we conclude that patients treated with cisplatin and paclitaxel had more tumor-infiltrating T-cell modulation than patients treated with cisplatin monotherapy. These findings enhance our understanding of the immune-modulating effect of chemotherapy and warrant future combination of the standard-of-care therapy with immunotherapy to improve clinical outcome in patients with cervical cancer.

**Electronic supplementary material:**

The online version of this article (10.1007/s00262-019-02412-x) contains supplementary material, which is available to authorized users.

## Introduction

Cervical cancer is the fourth most frequent cancer in women, accounting for 7.5% of cancer-related deaths worldwide [1]. The disease is caused by persistent infection with human papillomavirus, mostly high-risk types 16 and 18 [2]. The standard of care depends on tumor stage and size, and consists of either primary surgery or concurrent chemoradiation. Unfortunately, this treatment approach does not control loco-regional disease in all patients [3]. Neoadjuvant chemotherapy (NACT) before surgery currently has a place in clinical trials and for specific cases, but it is not yet considered standard care [4–6].

In the chemotherapeutic management of cervical cancer, platinum compounds (mainly cisplatin) are often the treatment of choice [7]. Cisplatin affects DNA replication and transcription via the crosslinking of DNA strands, suppressing the growth and proliferation of tumor cells [8]. Paclitaxel is another frequently used cytostatic chemotherapy type [7]. This drug inhibits the mitosis of tumor cells by stabilizing guanosine diphosphate-bound tubulin in microtubules [8]. In a phase III clinical trial in patients with advanced stage or recurrent cervical cancer, the combination of cisplatin and paclitaxel was superior to treatment with cisplatin alone with respect to the objective response rate and progression-free survival, but without improvement in overall survival [9]. Regrettably, resistance to chemotherapy is one of the major factors limiting therapeutic efficacy and influencing clinical outcome [10], which is likely the cause of failure to control loco-regional disease in patients with late-stage cervical cancer [3].

Fortunately, chemotherapy can also induce anti-tumor immunity through the modulation of immune cells present in the tumor microenvironment (TME) [8]. This has been demonstrated in cervical cancer. Meng et al. compared the number of CD8^+^ T cells and PD-L1 and PD-1 checkpoint expression in cervical tumor samples from patients treated with or without NACT; both CD8^+^ T-cell numbers and checkpoint expression were higher in patients previously treated with NACT [11]. Liang et al. showed no increase in CD8^+^ T cells in the cervical TME after NACT, but they found less FoxP3 expression after NACT [12]. Furthermore, enhancing effects on both the myeloid and T-cell composition have been found in blood samples from cervical cancer patients [13] and tumor-draining lymph nodes [14] after neoadjuvant (chemo)therapy.

Extensive information on the actual effect of NACT on the TME is still lacking. Here, for the first time, we studied the modulating effect of NACT on the numbers and phenotypes of tumor-infiltrating CD4^+^ and CD8^+^ T cells in primary cervical tumors. This pilot study improves our understanding of the TME and, specifically, the effect of NACT on the TME. The first clinical trials combining regular chemotherapy with immunotherapy have already shown promising results in various tumor types [15], and this may also be applied in patients with cervical cancer to improve therapeutic outcomes.

## Materials and methods

### Patient cohort

Formalin-fixed, paraffin-embedded (FFPE) matched pre- and post-NACT tumor specimens (*n *= 26) were retrospectively selected from 13 patients with squamous-cell cervical cancer at Leiden University Medical Center. All patients had large tumors (≥ 4 cm) and underwent NACT according to local protocol with cisplatin (*n *= 6) or a combination regimen including cisplatin and paclitaxel (*n *= 7) prior to surgery (mean 31.1 days prior). Treatment in the cisplatin group consisted of intravenous cisplatin (50 mg/m^2^) repeated every 3 weeks for the duration of six cycles. In the combination group, patients received intravenous paclitaxel (135 mg/m^2^) over 25 h on day 1 and intravenous cisplatin (50 mg/m^2^) at a rate of 1 mg/minute on day 2. This cycle was repeated every 3 weeks for the duration of six cycles. One out of six patients did not complete cisplatin treatment due to hypertension and progressive disease. Two out of seven patients completed cisplatin and paclitaxel treatment; data were missing on NACT completion for three patients, one patient did not complete treatment due to kidney failure, and one patient received carboplatin in combination with paclitaxel for a few cycles, see Table 1 for patient characteristics.

**Table 1:** Patient characteristics

Characteristic	NACT treatment		*P* value
	Cisplatin^a^	Cisplatin + paclitaxel^b^	
Number of patients	6	7	–
Age, years			
Mean	40.3	27.3	0.001*
Min	35	21	
Max	45	32	
FIGO stage^c^			
IB1–IB2	0	1	
IB2	6	4	
IB2–IIB	0	1	
IIB	0	1	0.559^#^
Tumor size pre-NACT			
Mean, cm	5.45	4.30	0.035*
Tumor size post-NACT			
Mean, cm	4.50	1.09	0.005*
Surgery after NACT	Radical hysterectomy	Radical trachelectomy or hysterectomy	–
Pathological response^d^			
Responder	2	6	
Non-responder	4	1	0.103^#^
Clinical response^e^			
Responder	1	6	
Non-responder	5	1	*0.029*^#^

Italics indicate significant *P* values

^a^1 out of the 6 patients did not complete cisplatin treatment due to hypertension and progressive disease

^b^Only 2 of the 7 patients completed cisplatin and paclitaxel treatment. Data on NACT completion are missing for 3 patients, 1 patient did not complete treatment due to kidney failure, and 1 patient received carboplatin in combination with paclitaxel for a few cycles

^c^FIGO stage: stage according to International Federation of Gynecology and Obstetrics

^d^Pathological response defined as responder: no residual tumor (complete response), minimal residual tumor (individual tumor cells and nests < 2 mm, optimal partial response), and easily identifiable tumor (sheets and nests > 2 mm), but also areas with response (suboptimal partial response). Non-responder defined as: no identifiable response

^e^Clinical response defined as responder: no residual tumor left upon medical examination/imaging (complete response) and at least a 30% decrease in the maximum size of the tumor (partial response). Non-responder defined as: < 30% decrease and < 20% increase in maximum tumor size (stable disease) and > 20% increase in maximum tumor size (progressive disease)

*P value was calculated by the Mann–Whitney *U* test

^#^*P* value measured by the Fisher’s exact test

### Multiplex immunohistochemistry

Multiplexed tyramide signal amplification (TSA) immunofluorescent staining was performed on pre- and post-NACT cervical tumor samples to phenotype and enumerate different tumor-infiltrating T-cell populations using the OPAL 7-color fluorescence immunohistochemistry (IHC) Kit (Perkin Elmer, USA), see Supplementary Table 1 for the studied T-cell phenotypes.

Sections (4-μm-thick) were cut from the FFPE blocks of the cervical tumors and control samples, including tonsil and cervical metastatic lymph node. Slides were deparaffinized, rehydrated, and endogenous peroxidase activity blocked with 0.03% H_2_O_2_ in methanol for 20 min. An extra fixation step was included for 20 min with 10% neutral buffered formalin (Leica Biosystems, Germany). Antigen retrieval was carried out by placing the slides in a plastic tray and heating in 0.05% ProClin300/Tris–EDTA buffer at pH 9.0 in an 800 W standard microwave at 100% power until the boiling point, followed by 15 min at 30% power. The following primary antibodies were used: 1:1000 mouse anti-CD8 (4B11, Novocastra, Wetzlar, Germany), 1:750 rabbit anti-CD3 (Abcam, Cambridge, UK), 1:750 mouse anti-FoxP3 (236A/E7, Abcam, Cambridge, UK), 1:1000 rabbit anti-Tbet (H-210, Santa Cruz, Dallas, Texas, USA), and 1:500 rabbit anti-Ki67 (SP6, Abcam, Cambridge, UK). The following steps were repeated for each primary antibody. The slides were allowed to cool and blocked with Normal Antibody Diluent (Immunologic, the Netherlands). The slides were then incubated with primary antibody diluted in Normal Antibody Diluent for 30 min at room temperature (RT) and 30 rpm on a shaker, followed by incubation with the broad spectrum HRP from the SuperPicture Polymer Detection Kit (Life Technologies, USA) for 20 min at RT and 30 rpm. Next, the slides were incubated with Opal TSA fluorochromes (Opal540, Opal570, Opal620, Opal650, and Opal690) diluted in amplification buffer (all provided by the OPAL 7-color fluorescence IHC Kit) for 10 min at RT and 30 rpm. The primary and secondary antibody complex was stripped by either microwave treatment with 0.05% ProClin300/Tris–EDTA buffer at pH 9.0 (for CD8 and CD3) or using a denaturing solution kit (BioCare Medical) for 5 min at RT and 30 rpm (for FoxP3, Tbet, and Ki67). Finally, DAPI working solution (provided by the OPAL 7-color fluorescence IHC Kit) was applied for 5 min at RT and the slides mounted under coverslips with ProLong Diamond anti-fade mounting medium (Life Technologies, USA).

Multiplex TSA IHC was optimized by testing all antibodies individually using both chromogenic 3,3′-diaminobenzidine as previously described [16] and the TSA visualization method to test different orders, incubation times, and antibody dilutions.

Tonsil and metastatic cervical lymph node samples were used as positive controls for all of the markers. A negative control was carried out by following the complete protocol but omitting primary antibody incubation.

### Imaging and quantification

Six-color multiplex staining was visualized by a confocal laser scanning TCS SP8 microscope (Leica, Germany) and tilescan images (3 × 3, 40× oil objective with 1.3 NA) generated and viewed using LAS AF Lite software (Leica, Germany). Tagged image file formats were used for quantification analysis in TissueStudio^®^ (Definiens, Germany). Using self-learning algorithms in TissueStudio^®^, tissue detection and segmentation (stroma vs. tumor) were carried out and nucleus and cell segmentation obtained prior to co-expression marker analysis. Thresholds were set per marker and the co-expression of three different markers in both tumor and stromal areas analyzed and denoted as the number of positive cells per mm^2^.

### Statistical analysis

Heatmap and cluster analyses were carried out using the function heatmap.2 in RStudio Version 1.1.423 (RStudio, USA). Statistical analyses were performed using R version 3.4.4 [17] and GraphPad Prism 5 (GraphPad Software, USA). The significance between pre- and post-NACT samples was calculated by the Wilcoxon signed rank test. *P* values < 0.05 were considered significant.

## Results

### Patient cohort

Patients with large cervical tumors (4–7 cm) were treated with cisplatin (*n *= 6) to reduce tumor volume to allow radical surgery instead of chemoradiation or were treated with cisplatin and paclitaxel (*n *= 7) to allow for fertility preservation using a less radical surgical approach. Patients treated with neoadjuvant cisplatin only were older (40.3 vs. 27.3 years old, *P *= 0.001), manifested larger tumors pre-NACT (5.45 vs. 4.30 cm, *P *= 0.035) and post-NACT (4.50 vs. 1.09 cm, *P *= 0.005), and had a poorer clinical response (1/6 vs. 6/7 patients, *P *= 0.029; Table 1).

### Tumor-infiltrating T-cell subset analysis on pre- and post-NACT cervical cancer

**Fig. 1:**
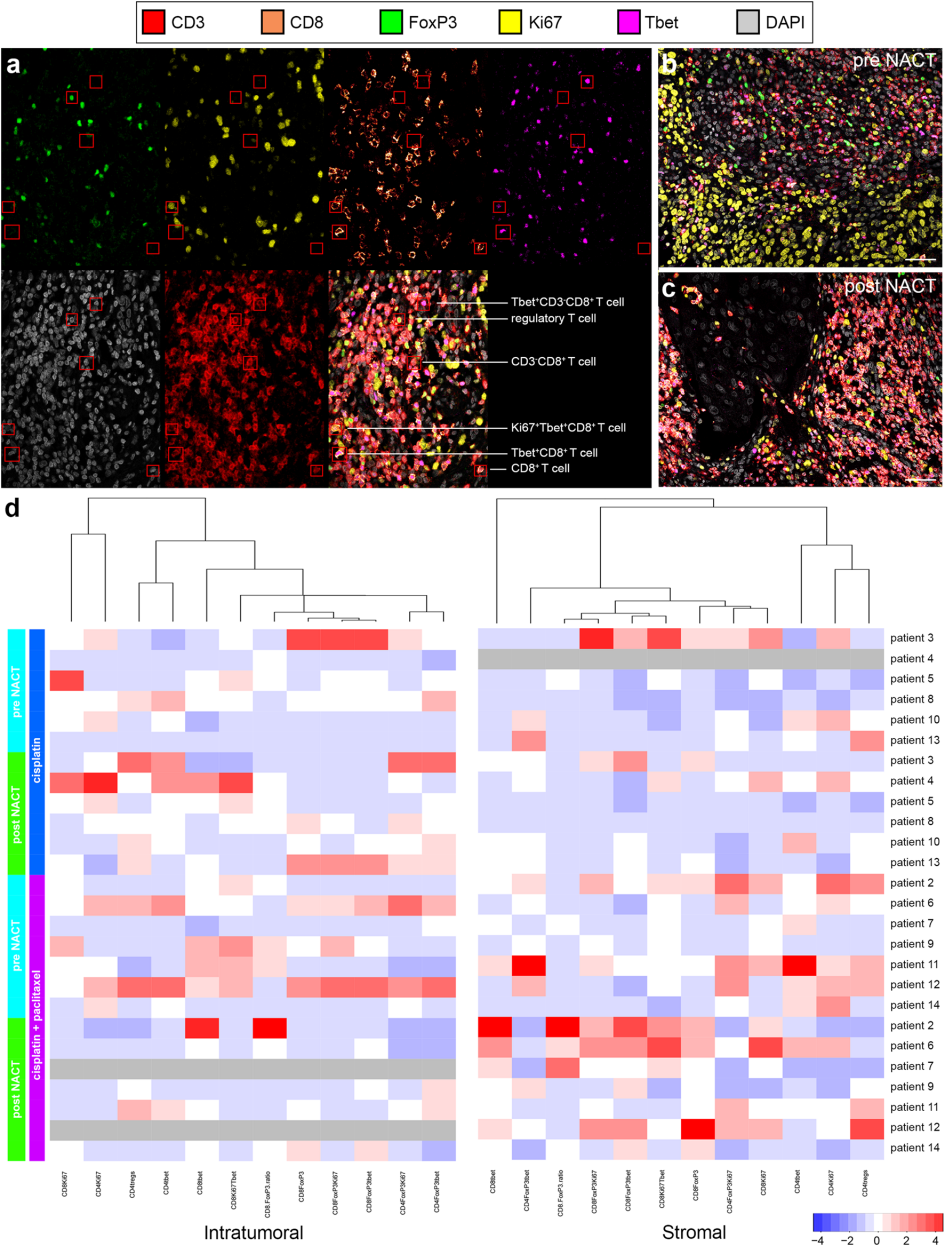
T-cell subset analysis of pre- and post-neoadjuvant chemotherapy-treated cervical cancer. **a** Example of the identification of various T-cell subsets using multiplex IHC: Tbet^+^CD8^−^ T cells, (CD3^+^CD8^−^Tbet^+^); regulatory T cells (CD3^+^CD8^−^FoxP3^+^); CD8^−^ T cells (CD3^+^CD8^−^); proliferating Tbet^+^CD8^+^ T cells (CD8^+^Ki67^+^Tbet^+^); Tbet^+^CD8^+^ T cells (CD3^+^CD8^+^Tbet^+^); and CD8^+^ T cells (CD3^+^CD8^+^). Representative six-color multiplex image of a matched **b** pre- and **c** post-neoadjuvant chemotherapy (NACT) cervical tumor sample. **d** Heatmap showing that most changes in tumor-infiltrating T-cell rates manifested in the stromal compartment of tumors in patients treated with cisplatin and paclitaxel. Patients 7 and 12 had a complete response; therefore, no data can be shown for the intratumoral compartment after NACT (grey). For patient 4, no reliable data were available for the stromal compartment (grey)

We phenotyped and compared various tumor-infiltrating T-cell subsets using CD3, CD8, FoxP3, Tbet, and Ki67 as markers in multiplex IHC on pre- and post-NACT tumor samples from patients with cervical cancer. Various T-cell subsets used in the quantification co-expression analysis are shown in Fig. 1a. A representative six-color multiplex image of matched pre- and post-NACT cervical tumor samples is shown in Fig. 1b, c. An overview of the studied T-cell subsets and corresponding percentages is given in Supplementary Table 1.

**Fig. 2:**
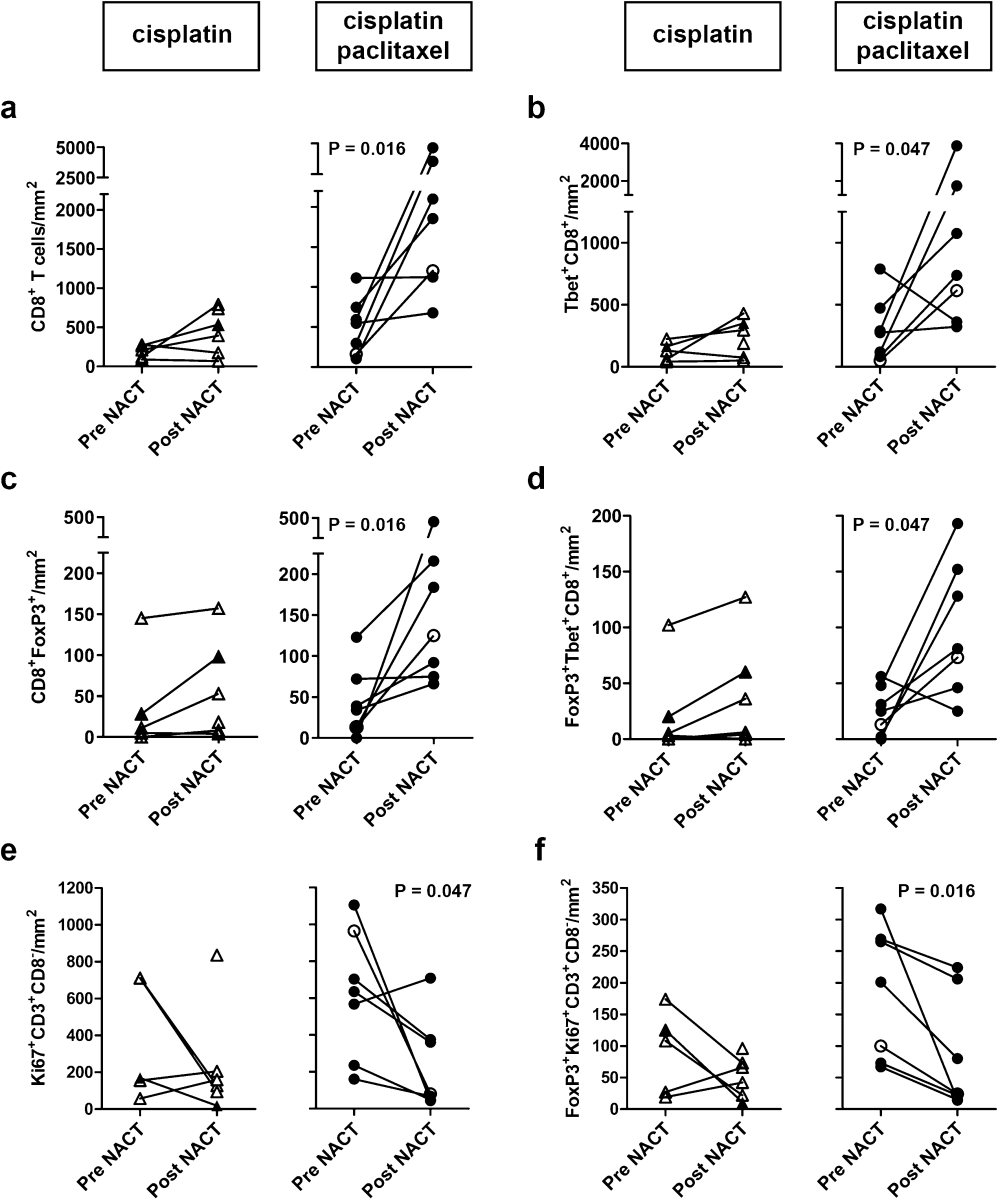
Alterations in tumor-infiltrating T-cell rates after neoadjuvant chemotherapy. Tumor-infiltrating T cells were quantified and analyzed for phenotype in matched pre- and post- neoadjuvant chemotherapy (NACT) tumor samples from patients with cervical cancer. **a** CD8^+^ T-cell numbers, **b** Tbet^+^CD8^+^ T cells, **c** FoxP3^+^CD8^+^ T cells, **d** FoxP3^+^Tbet^+^CD8^+^ T cells, **e** Ki67^+^CD8^−^ T cells, and **f** FoxP3^+^Ki67^+^CD8^−^ T cells per square millimeter in patients treated with cisplatin alone (triangles, left) and in patients treated with cisplatin and paclitaxel (circles, right). Open triangles and open circles represent patients who lacked a clinical response. In the cisplatin only group, no reliable data were available pre-NACT for one patient. *P* values were calculated by the Wilcoxon signed rank test

Before the start of the treatment, no significant differences were found in T-cell subset frequencies between the two different treatment groups. Patients treated with the combination cisplatin and paclitaxel regimen had therapy-induced T-cell modulation, whereas little to no effect on T-cell rates was seen in patients treated with cisplatin only. Notably, these alterations in T-cell frequencies were observed only in the stromal compartment of the tumors (Figs. 1d, 2).

We observed increased levels of cytotoxic CD8^+^ T cells (*P *= 0.016), Tbet^+^CD8^+^ T cells (*P *= 0.047), FoxP3^+^CD8^+^ T cells (*P *= 0.016), and FoxP3^+^Tbet^+^CD8^+^ T cells (*P *= 0.047) after treatment with the combination cisplatin and paclitaxel regimen (Fig. 2a–d). These patients also tended to have a higher CD8^+^/regulatory T-cell (Treg) ratio after NACT (*P *= 0.078).

Chemotherapy is known to have a direct negative effect on rapidly dividing, proliferating cells. We observed a decrease in proliferating cells in the stromal compartment, but only in patients treated with the combination of cisplatin and paclitaxel, with lower levels of proliferating CD4^+^ T cells (identified as CD3^+^CD8^−^Ki-67^+^; *P *= 0.047; Fig. 2e) and proliferating Tregs (identified as CD3^+^CD8^−^FoxP3^+^Ki-67^+^; *P *= 0.016; Fig. 2f) after NACT.

## Discussion

In this era of rapidly evolving knowledge on the tumor immune landscape and the growing potential of targeted therapies in combination with traditional treatments, conventional chemotherapy has clearly been shown to have immunoregulatory properties. For example, several clinical trials in melanoma, lung cancer, and bladder cancer have demonstrated improved patient outcomes after chemotherapy in combination with immunotherapy [15]. In cervical cancer, information is lacking on the effect of NACT on the local immune microenvironment. This study is the first to use multiplex immunohistochemistry on matched pre- and post-NACT cervical tumor samples to analyze and compare functionally divergent tumor-infiltrating T-cell subpopulations. This novel technique allows for comprehensive phenotyping of immune infiltrate in the context of tissue texture, even in limited tissue-like small biopsies, using an automated scoring algorithm.

In this study, we demonstrated that patients treated with the combination regimen consisting of cisplatin and paclitaxel had therapy-induced T-cell modulation, whereas little to no effect on T-cell infiltration rates was seen in patients treated only with cisplatin. Although clinico-pathological differences in age and tumor size were found between the two treatment groups, our observations suggest that the combination regimen has more potential to induce immunogenic tumor cell death and release NACT-induced tumor antigens that are taken up by the innate immune system, resulting in tumor-specific CD8^+^ T-cell activation and expansion. In an ovarian cancer xenograft mouse model, cisplatin and paclitaxel were shown to synergistically generate strong tumor-specific CD8^+^ T-cell responses [18]. This finding is in concordance with the clinical observation in patients with cervical cancer that response rates, both objective and complete, and progression-free survival were better for patients receiving both cisplatin and paclitaxel than patients receiving cisplatin alone, both in this study and others [9].

We observed higher NACT-induced rates of cytotoxic CD8^+^ T-cell expression of Tbet and/or FoxP3 after cisplatin and paclitaxel treatment compared to cisplatin only. Tbet expression may be a sign of interferon-γ production and has been shown to correlate positively with disease outcome in cervical cancer [19]. The role of FoxP3-expressing CD8^+^ T cells in cervical cancer is controversial. Battaglia et al. demonstrated that CD8^+^FoxP3^+^ T cells are able to efficiently inhibit the proliferation of both CD4^+^ and CD8^+^ T cells in cervical tumor-draining lymph nodes [20]. Recently, our group showed that these cells have an exhausted phenotype (high expression of checkpoint molecules CTLA-4, PD-1, and TIM-3) with superior effector functions [21]. However, the increased rate of FoxP3 and Tbet co-expression by CD8^+^ T cells after NACT observed in the current study may be indicative of a positive role in anti-tumor immune responses.

Furthermore, we measured a decrease in proliferating (Ki67^+^) CD4^+^ T cells and Tregs only in patients treated with the combination cisplatin and paclitaxel regimen. This finding is consistent with chemotherapy in general having a direct negative effect on rapidly dividing, proliferating cells, mostly tumor cells, with the synergistic effect of cisplatin and paclitaxel probably being more effective. Though some chemotherapeutic regimens (e.g., cyclophosphamide and gemcitabine) can reduce the number and function of Tregs [8], we did not observe changes in non-proliferating Tregs after neoadjuvant cisplatin or cisplatin plus paclitaxel treatment. Similar findings were found by Fattorossi et al. [14] in the tumor-draining lymph nodes of patients with cervical cancer treated with neoadjuvant cisplatin. Though one study of cervical cancer reported a significant decrease in FoxP3^+^ T cells in the TME after NACT [12], they stained only a single marker, FoxP3, for IHC and did not identify these FoxP3-expressing cells using other markers. Furthermore, the included group of patients had different tumors based on histological subtypes and were treated with different types of NACT [12].

The most notable alterations in tumor-infiltrating T-cell rates after NACT were found in the stromal compartment, as opposed to the intratumoral compartment. Most immune cells accumulate in the stromal compartment (peritumoral), as the tumor possesses a variety of mechanisms (i.e., checkpoint molecule expression) that contribute to the immunosuppressive activity leading to lower or absent tumor infiltration [22]. Meng et al. [11] reported recently on the increased expression of checkpoint molecules PD-1 and PD-L1 in cervical tumor tissue from patients previously treated with NACT.

Though the current study has limited power to find significant effects due to the sample size, some of our results are consistent with what is already known about cervical cancer, such as increased levels of CD8^+^ T cells after NACT [11, 12]. In addition, we found interesting new trends, such as the increase in specific CD8^+^ T-cell subsets expressing Tbet.

Additional studies on large and homogeneous patient cohorts are needed to examine the effect of NACT on immune checkpoint molecule expression. The ultimate goal would be to attempt to interrupt the chemoresistant niche, possibly through a combinatorial approach including immunotherapy (e.g., checkpoint blockade) to improve anti-tumor immune responses and control loco-regional disease in cervical cancer patients.

In conclusion, this pilot study demonstrates that neoadjuvant treatment with synergizing cisplatin and paclitaxel more effectively enhances cytotoxic T-cell modulation compared to cisplatin monotherapy. Further studies are needed to increase the power of our preliminary findings and should focus on tumor cells, dendritic cells, macrophages, and myeloid-derived suppressor cells and checkpoint expression in the cervical TME to unravel the interplay with T cells. In the recently closed EORTC 55994 study, in which NACT followed by surgery was compared to concurrent chemoradiation in a large European patient cohort (NCT00039338), we will perform extensive TME analyses and link the results to clinical characteristics and patient survival. Similar analyses have been reported in ovarian cancer [23] and lung cancer [24].

As the first clinical trials on combining chemotherapy with immunotherapy have already shown promising results in various tumor types [15], it is crucial, given the poor outcome of advanced stage cervical cancer, to unravel chemotherapy-induced TME immunomodulation and determine what type of immunotherapy could improve anti-tumor immune responses and, ultimately, patient prognosis.

## Electronic supplementary material

Below is the link to the electronic supplementary material.

Supplementary material 1 (PDF 276 kb)

## Abbreviations

FFPE: Formalin-fixed, paraffin-embedded
IHC: Immunohistochemistry
NACT: Neoadjuvant chemotherapy
RT: Room temperature
TME: Tumor microenvironment
TSA: Tyramide signal amplification
