# Supplementary material for: Neoadjuvant cisplatin and paclitaxel modulate tumor-infiltrating T cells in patients with cervical cancer
Source: Cancer Immunol Immunother. 2019 Oct 15;68(11):1759–67. doi: 10.1007/s00262-019-02412-x (PMC6851216; doi:10.1007/s00262-019-02412-x)
Supplement: Supplementary file 1 — Supplementary material 1 (PDF 276 kb) [file 262_2019_2412_MOESM1_ESM.pdf]

Supplementary Table 1. T-cell phenotype and numbers before and after NACT in primary tumor samples from patients with cervical cancer.

			Cisplatin (*n*=6)		Cisplatin + paclitaxel (*n*=7)	
**Target population**	**Phenotype**	**Area**	**Pre-NACT**	**Post-NACT**	**Pre-NACT**	**Post-NACT**
T cells	$CD3^+$	Stromal	2365.2 ± 697.8	2969.5 ± 895.3	3833.3 ± 694.3	5189.6 ± 876.2
		Intratumoral	1092.7 ± 141.7	1892.1 ± 501.1	1308.0 ± 315.6	1310.8 ± 223.4
$CD8^-$ T cells	$CD3^+CD8^-$	Stromal	1673.5 ± 657.7	2102.2 ± 765.5	2660.6 ± 505.4	2388.8 ± 568.6
		Intratumoral	612.4 ± 66.6	1111.3 ± 375.8	795.7 ± 236.5	721.9 ± 158.3
$CD8^+$ T cells	$CD3^+CD8^+$	Stromal	192.6 ± 38.0	449.6 ± 119.8	509.0 ± 134.5	2166.3 ± 544.1*
		Intratumoral	358.3 ± 173.8	526.3 ± 193.1	330.0 ± 81.6	453.6 ± 145.6
Tregs$^{\#}$	$CD3^+CD8^-FoxP3^+$	Stromal	461.3 ± 173.1	361.4 ± 52.5	566.5 ± 108.4	488.2 ± 198.0
		Intratumoral	105.7 ± 24.7	238.3 ± 58.0	166.1 ± 74.0	123.0 ± 65.4
$CD8^+$ T cell/Treg ratio		Stromal	1.0 ± 0.6	1.2 ± 0.2	1.2 ± 0.4	16.8 ± 8.4
		Intratumoral	4.8 ± 2.7	3.5 ± 1.5	8.4 ± 4.3	29.3 ± 24.7
$FoxP3^+CD8^+$ T cells	$CD3^+CD8^+FoxP3^+$	Stromal	37.7 ± 27.3	56.2 ± 24.9	41.9 ± 16.2	177.2 ± 55.1*
		Intratumoral	16.3 ± 11.6	16.3 ± 7.9	16.3 ± 7.5	12.3 ± 4.1
Proliferating $CD8^-$ T cells	$CD3^+CD8^-Ki67^+$	Stromal	360.4 ± 144.3	239.8 ± 121.9	624.2 ± 131.2	240.2 ± 95.5*
		Intratumoral	302.9 ± 67.8	464.1 ± 233.4	403.7 ± 99.4	107.7 ± 28.4
Proliferating $CD8^+$ T cells	$CD3^+CD8^-Ki67^+$	Stromal	106.0 ± 57.8	85.5 ± 37.4	143.2 ± 36.7	168.7 ± 53.0
		Intratumoral	299.1 ± 171.3	261.7 ± 138.4	252.9 ± 67.2	60.4 ± 23.1
Proliferating cells	$Ki67^+(CD3^-CD8^-)$	Stromal	1065.8 ± 241.8	599.1 ± 252.7	2201.6 ± 446.8	451.3 ± 199.2*
		Intratumoral	4534.0 ± 1159.0	4456.5 ± 959.5	7052.2 ± 996.6	1795.3 ± 1168.9
Proliferating Tregs	$CD8^-FoxP3^+Ki67^+$	Stromal	90.7 ± 29.7	51.7 ± 13.3	184.7 ± 39.3	83.6 ± 35.0*
		Intratumoral	52.5 ± 12.2	100.7 ± 26.2	83.9 ± 33.8	34.1 ± 15.2
Proliferating $FoxP3^+CD8^+$ T cells	$CD8^+FoxP3^+Ki67^+$	Stromal	23.0 ± 21.5	8.5 ± 4.1	13.6 ± 6.3	31.2 ± 10.1
		Intratumoral	9.1 ± 5.9	7.6 ± 4.0	10.1 ± 4.5	2.1 ± 0.7
$Tbet^+CD8^-$ T cells	$CD3^+CD8^-Tbet^+$	Stromal	549.7 ± 136.4	606.5 ± 131.5	1072.2 ± 272.6	583.7 ± 113.4
		Intratumoral	205.4 ± 47.5	333.5 ± 76.5	278.6 ± 92.1	178.1 ± 48.1
$Tbet^+CD8^+$ T cells	$CD3^+CD8^+Tbet^+$	Stromal	124.6 ± 33.7	231.9 ± 62.3	297.1 ± 98.9	1246.2 ± 474.7*
		Intratumoral	101.0 ± 25.5	151.0 ± 52.9	165.4 ± 38.9	222.9 ± 94.0
$Tbet^+$ cells	$Tbet^+(CD3^-CD8^-)$	Stromal	230.1 ± 66.1	317.1 ± 65.2	621.9 ± 228.7	325.4 ± 93.1*
		Intratumoral	193.5 ± 64.1	429.7 ± 190.6	159.7 ± 19.9	212.6 ± 74.5
$Tbet^+$ Tregs	$CD8^-FoxP3^+Tbet^+$	Stromal	223.8 ± 78.8	152.3 ± 20.6	299.6 ± 98.2	116.2 ± 37.7
		Intratumoral	56.0 ± 17.9	105.2 ± 26.1	74.9 ± 32.1	46.1 ± 24.2
$FoxP3^+Tbet^+CD8^+$ T cells	$CD8^+FoxP3^+Tbet^+$	Stromal	26.1 ± 19.2	38.8 ± 20.1	25.0 ± 8.3	99.6 ± 22.7*
		Intratumoral	10.3 ± 7.4	9.0 ± 5.2	11.2 ± 5.3	7.0 ± 2.1
Proliferating $Tbet^+CD8^+$ T cells	$CD8^+Ki67^+Tbet^+$	Stromal	61.7 ± 36.5	38.8 ± 9.5	58.7 11.9	82.3 25.5
		Intratumoral	61.4 ± 19.2	85.5 ± 42.9	112.8 ± 24.7	18.3 ± 3.0

Data are presented as the mean number of cells/mm^2 ± standard error of the mean; $^{\#}$Tregs: regulatory T cells; *P = 0.01- 0.05 as calculated by the Wilcoxon signed rank (paired) test.
